# Development of clinical reasoning in an undergraduate medical program at a Brazilian university: Desenvolvimento do raciocínio clínico na graduação médica em uma universidade brasileira

**DOI:** 10.1590/1516-3180.2015.00080108

**Published:** 2015-12-08

**Authors:** Alexandre Roberti, Maria do Rosário Ferraz Roberti, Edna Regina Silva Pereira, Celmo Celeno Porto, Nilce Maria da Silva Campos Costa

**Affiliations:** I MD, MSc. Assistant Professor, Medical School, Universidade Federal de Goiás (UFG), Goiânia, Goiás, Brazil.; II MD, PhD. Adjunct Professor, Medical School, Universidade Federal de Goiás (UFG), Goiânia, Goiás, Brazil.; III MD, PhD. Emeritus Professor, Medical School, Universidade Federal de Goiás (UFG), Goiânia, Goiás, Brazil.; IV RD, PhD. Associate Professor of Nutrition, Universidade Federal de Goiás (UFG), Goiânia, Goiás, Brazil.

**Keywords:** Qualitative research, Students, medical, Education, medical, Curriculum, Cognition

## Abstract

**CONTEXT AND OBJECTIVE:**

The cognitive processes relating to the development of clinical reasoning are only partially understood, which explains the difficulties in teaching this skill in medical courses. This study aimed to understand how clinical reasoning develops among undergraduate medical students.

**DESIGN AND SETTING:**

Quantitative and qualitative exploratory descriptive study conducted at the medical school of Universidade Federal de Goiás.

**METHODS:**

The focus group technique was used among 40 students who participated in five focus groups, with eight students from each year, from the first to fifth year of the medical school program. The material was subjected to content analysis in categories, and was subsequently quantified and subjected to descriptive statistical analysis and chi-square test for inferential statistics.

**RESULTS:**

The content of the students’ statements was divided into two categories: clinical reasoning - in the preclinical phase, clinical reasoning was based on knowledge of basic medical science and in the clinical phase, there was a change to pattern recognition; knowledge of basic medical science - 80.6% of the students recognized its use, but they stated that they only used it in difficult cases.

**CONCLUSION:**

In the preclinical phase, in a medical school with a traditional curriculum, clinical reasoning depends on the knowledge acquired from basic medical science, while in the clinical phase, it becomes based on pattern recognition.

## RESUMO

**CONTEXTO E OBJETIVO:**

Tem-se compreensão parcial dos processos cognitivos relacionados ao desenvolvimento do raciocínio clínico, o que justifica as dificuldades no ensino dessa competência nos cursos de medicina. Este estudo tem como objetivo compreender como se desenvolve o raciocínio clínico em acadêmicos de medicina.

**TIPO DE ESTUDO E LOCAL:**

Pesquisa descritiva exploratória quantitativa e qualitativa, realizada na Faculdade de Medicina da Universidade Federal de Goiás.

**MÉTODOS:**

A técnica de grupos focais foi utilizada entre 40 acadêmicos, que participaram de cinco grupos focais, com oito acadêmicos de cada ano, do primeiro ao quinto ano do curso médico. O material foi submetido a análise de conteúdo por categorias, posteriormente quantificado e submetido a análise estatística descritiva e teste de qui-quadrado para estatística inferencial.

**RESULTADOS:**

O conteúdo das falas dos participantes foi dividido em duas categorias: raciocínio clínico - na fase pré-clínica, o raciocínio clínico é baseado no conhecimento das disciplinas básicas e, na fase clínica, há uma mudança para o reconhecimento de padrões; conhecimento das disciplinas básicas - 80,6% percebem sua utilização, porém assinalam que o usam apenas em casos difíceis.

**CONCLUSÃO:**

Na fase pré-clínica, em uma escola médica com currículo tradicional, o raciocínio clínico é dependente dos conhecimentos adquiridos nas disciplinas básicas e, na fase clínica, passa para o reconhecimento de padrões.

## INTRODUCTION

Medical training is based on construction of a cognitive structure.^1^^,^^2^ The cognitive processes relating to clinical reasoning are only partially understood.^3^^,^^4^^,^^5^^,^^6^ The ability to memorize the contents of both basic science and large sets of clinical cases does not provide experience.^7^ Rather, experience depends on the ability to memorize the content together with supervised professional practice.^1^^,^^8^

Two fundamental approaches have been recognized for reasoning: intuitive and analytical reasoning,^1^^,^^5^^,^^9^^,^^10^^,^^11^^,^^12^ which present different components. Intuitive reasoning involves pattern recognition (through categorization, a theory of disease scripts or a mental models theory), intuition and heuristics; while analytical reasoning involves hypothetical-deductive and probabilistic approaches.

Pattern recognition implies speed. The clinical reasoning of experienced students does not involve testing hypotheses in common situations. The theory of pattern recognition aims to explain how human beings understand the world.^13^ Memory involves encoding cognitive structures.^14^ A pattern determines what is normal and what is a variation of the norm.^13^^,^^15^

Medical students recognize signs and symptoms in a patient context when they perform their activities.^7^ These perceptions activate recognition of disease patterns, with which they interpret the information about the characteristics of that situation.^9^^,^^11^^,^^13^^,^^15^^,^^16^ The patterns present limited knowledge about the causal mechanisms but a large amount of information about the signs and symptoms of diseases. By applying these cognitive structures, the students quickly generate diagnoses for routine problems.^17^

Intuition can change decisions and lead to better performance than analytical deliberation. Students are advised not to trust their intuition, so as to avoid reasoning errors. Although this process is present and influences physician decision-making, it represents only part of the whole process.^18^

Heuristics is the process that aims to simplify complex reasoning relating to diagnoses that meet the established requirements. These cognitive shortcuts depend on previous knowledge. Heuristics is quick decision-making.^19^

The hypothetical-deductive approach can also be called the critical method, or Popper’s method of trial and error. To solve problems, the students use a cognitive method similar to the scientific method, or approaches used by detectives in addressing a crime.^20^

Several hypotheses are generated when a student addresses a real-world problem. Each hypothesis is sequentially tested, in order to be confirmed or eliminated, and then the final decision is made.^9^^,^^11^ Therefore, knowledge of basic medical science is important for establishing the cognitive structures and a relationship between the pathophysiology of the disease and the clinical characteristics of patients.^1^^,^^7^^,^^21^ This probabilistic approach implies that the analysis of clinical problems should be based on a Bayesian approach, i.e. systematic use of the Bayes theorem in which the post and pre-test probabilities (i.e. the prevalence of the disease) are correlated. The students may use health statistics in association with their initial clinical experience.^22^ In fact, only a small percentage of students use a Bayesian approach, and most of them use an informal method of data review.

A student’s level of knowledge changes with practice.^5^^,^^11^^,^^23^ In diagnosing common problems, experienced students tend to use quick and automatic reasoning (pattern recognition).^5^^,^^14^^,^^21^^,^^24^ In cases of more complex problems in which there is no recognized pattern,^15^ an analytical/reflective approach (hypothetical-deductive method) that uses the stored knowledge of basic science is triggered. Automatic reasoning tends to be efficient in routine situations,^14^^,^^24^ but it can lead to mistakes when the problems are complex.^21^^,^^24^

The understanding of learning has advanced considerably over recent decades, thus affecting various teaching and learning strategies. Recently, not only the curricula but also the scenarios and strategies of teaching and learning have been restructured.^25^ Medical practice requires multiple skills,^26^ which include clinical reasoning.^6^ The difficulties in teaching this skill are due to lack of knowledge about its development.^5^ Knowledge of the process of developing clinical reasoning is a requirement for its comprehension and for improvement of medical training.

## OBJECTIVE

This study aimed to understand the development of the process used for clinical reasoning among first to fifth-year undergraduate students at a medical school with a traditional curriculum in a federal public institution of higher education in Brazil.

## METHODS

A cross-sectional, descriptive, exploratory, qualitative and quantitative study was conducted. The focus group technique was used to gather data. This study was approved by the UFG (Federal University of Goiás) Ethics Committee, under number 176/12.

The medical course that was the subject of this study had a traditional curriculum and was offered at a medical school located in the center-west of Brazil. Every year, 110 new undergraduate students begin a six-year course that is divided into a preclinical phase (two years) and a clinical phase (four years) that includes two years of supervised training.

The participants were recruited at the institution investigated. First to fifth-year undergraduate students older than 18 years who were enrolled in the medical course and who agreed to participate by signing a free and informed consent statement were invited to participate in the study. The following undergraduate students were excluded: those younger than 18 years; those with enrolment in the medical course that had not been regularized in the medical school’s office; those whose year of enrolment was not clear; those who refused to participate, fearing embarrassment that could occur through the study; and sixth-year undergraduate students, because they were receiving training away from the university campus.

**Chart 1.**
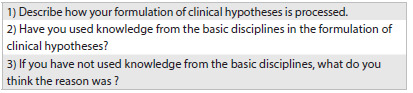
Key questions for the focus groups

A total of 40 undergraduate students were analyzed. They participated in five focus groups (eight students per academic year), with a focus group for each academic year of the course (first to fifth year). The focus groups were conducted in the classrooms, at predetermined times that avoided conflicts with the academic schedules, and lasted ninety minutes. A script with three questions (Chart 1) guided the discussion in the focus groups. The meetings were recorded and transcribed verbatim.

The material was evaluated by means of content analysis. The data were analyzed according to categories. The response units emerged within the categories, which described the main ideas discussed during the meetings. These ideas were quantified in each focus group. The quantification of response units was statistically analyzed.

Microsoft Excel 2007 and SPSS for Windows version 16.0 were used for statistical analysis. To evaluate differences in how clinical reasoning was organized and in perceptions of the use of basic medical science between the preclinical and clinical phases, the chi-squared test was used, with a significance level of 5% (P < 0.05).

## RESULTS

The content of the participants’ statements in the focus groups was divided into the categories of clinical reasoning and knowledge of basic medical science.

### Clinical reasoning

The majority of the undergraduate students in the preclinical phase developed their clinical reasoning based on knowledge acquired from basic medical science, using a line of reasoning based on knowledge about organs or body systems. This result was observed in 27/29 (93.1%) of the students’ statements in the preclinical phase. Some representative statements included “*We verify the symptoms, and then we observe the main system affected*” and “*I observed a group of symptoms and sought a system.*”

The clinical reasoning gradually changed in the statements of the undergraduate students during the clinical phase. The third and fourth-year undergraduate students still used the earlier knowledge of basic science, which was observed in 10/16 (62.5%) of the third-year undergraduate students’ statements and in 19/25 (76%) of the fourth-year undergraduate students’ statements: “*It comes from something that you already knew. The observations of signs and symptoms give you a sense of what organ or system is affected*”; “*I always reason from previous cases or something I have read*”; and “*I generate a diagnostic hypothesis based on knowledge of something that I have previously studied*.”

The results showed that the use of automatic reasoning developed over the third, fourth and fifth years of medical school. Thus, automatic reasoning was observed in 4/16 (25%), in 6/25 (24%) and in 11/14 (78.5%) of the statements of third, fourth and fifth-year undergraduate students, respectively: “*The first hypothesis that comes from automatic reasoning is important*”; “*It is an automatic thing, and you do not even notice it*”; and “*It just appeared.*”

In the preclinical phase, the clinical reasoning was based on previous knowledge obtained from basic medical science, while in the clinical phase, it became automatic. The difference in the frequencies of these types of statements was statistically significant between the phases (P < 0.001).

### Knowledge of basic medical science

A total of 50/62 (80.6%) of the students’ statements indicated that they used basic medical science in their clinical reasoning, while this was not observed in 12/62 (19.4%). Their statements included the following: “*From the standpoint of what is normal, something that is not happening normally should be an alteration of the normal; if you do not know what is normal, which is taught within basic medical science, you will not be able to understand what caused the disorder*”; and “*We use what we retain; things that are important.*” No statistically significant difference in the perception of the use of basic medical science for reasoning was observed between the preclinical and clinical phases (P = 0.95).

The statements of the students who did not use basic medical science included: “*I think we do not use most of the basic medical science*”; “*We do not consciously use it*”; “*The entire range of knowledge acquired over the first two years of the course are not used in practice*”; and “*I speak for myself, but I do not use it when I have to reason.*”

Students in all the years assessed stated that they always went back to the basic medical science in difficult cases: “*If the case is very difficult, I search for it in the areas of anatomy, biochemistry and histology to see if I can find some information*” (first-year); “*You go back to the basic medical science when you read a difficult case*” (third-year); “*I think about less common diseases; I reassess the systems. I use the basic medical science to interpret the findings*” (fourth-year); and “*We use the basic medical science when the case is difficult*” (fifth-year).

## DISCUSSION

### Clinical reasoning

The development of clinical reasoning observed in the present study is in agreement with the current theories of clinical reasoning: the hypothetical-deductive method and pattern recognition (scripts).^4^^,^^9^^,^^11^^,^^16^^,^^23^^,^^27^^,^^28^^,^^29^^,^^30^^,^^31^ The novelty of the present study is the better comprehension that it provides regarding the temporality of the students’ progress within the academic curriculum; i.e. the change in undergraduate medical students’ clinical reasoning stemming from their interaction with patients.

However, this study was not able to explain whether the migration from basic knowledge to pattern recognition occurs uniformly among students, or whether it is predominantly in the best students, because the students participated in the focus groups together and their statements do not have this information.

Many of our observations can be explained by the “Taxonomy of educational objectives” proposed by Bloom.^32^ There are three specific domains in this model: cognitive, affective and psychomotor. The cognitive and psychomotor domains involve acquisition of knowledge, intellectual development and physical ability. They include recognition of specific facts, standard procedures and concepts that stimulate intellectual development. The affective domain relates to values and attitudes.^33^

The cognitive domain comprises a) remembering: recognizing and reproducing ideas and contents; b) understanding: establishing a connection between the new information and previously acquired knowledge; c) applying: using a procedure in a specific or new situation; d) analyzing: understanding the interrelationship between the parts; e) evaluating: making judgments based on criteria; and f) creating: a new vision, a new solution.^33^

Through analyzing Bloom’s taxonomy, it can be inferred that the students in the preclinical phase can remember, understand and apply the knowledge acquired within basic medical science (organ systems). However, they have difficulty in analyzing and evaluating because they have not yet attended the courses that address clinical signs and symptoms. Therefore, hypothetical-deductive reasoning limited to knowledge of body systems is developed. Regarding the final category (creating), which was interpreted as diagnosis in the present study, it is very limited among students at this point of the medical course.

Also based on Bloom’s taxonomy, it can be inferred that the students of the clinical phase are already able to remember, understand, apply, analyze, evaluate and create; and that they gradually become able to complete a chain of hypothetical-deductive diagnostic reasoning or pattern recognition, depending on their experience.

However, it needs to be considered whether the migration from basic science to pattern recognition as the basis for reasoning might merely reflect the fact that students are progressively exposed to more clinically oriented content as the course advances.

It is believed that students will gradually assimilate the material and knowledge that they need through a mechanism known as knowledge integration.^2^^,^^7^^,^^34^ Integration of the knowledge obtained from basic medical science occurs due to repeated application of this knowledge within clinical practice environments, as an easier way to access the reasoning structures.^1^^,^^2^^,^^7^^,^^34^ Undergraduate students acquire the necessary biological knowledge during the preclinical phase of the medical course.^7^ During the clinical phase, they interact with patients and then apply the acquired knowledge. Thus, application of this knowledge associated with acquisition of practical knowledge begins to link the signs and symptoms to the diagnostic hypotheses. When applied to clinical reasoning, this link leads to integration of clinical and biomedical knowledge, thus concluding the process.^1^^,^^2^^,^^7^^,^^34^

Three stages in the development of clinical reasoning have been described: acquisition of knowledge of basic medical science; experience acquired through contact with patients; and integration of theoretical knowledge.^7^ Therefore, when third, fourth and fifth-year students start to come into contact with patients, they progressively exhibit the integration process and gradual formation of their scripts.^13^

The students’ statements demonstrated that during the preclinical and clinical phases, they followed different lines of reasoning to solve problems and that there was no single way to do this. Several cognitive actions occur, starting from when a clinical meeting begins: clinical knowledge is activated; scripts are mobilized and enriched; and integrated knowledge is accessed. These processes occur together and are controlled by meta-cognition, thus indicating that clinical reasoning is not a linear process but rather, a sequence of steps.^3^^,^^26^

### Knowledge of basic medical science

Our observations are in agreement with the current theories, which state that knowledge obtained from basic medical science is used in situations where pattern recognition (scripts) has not yet developed. In these cases, students use basic knowledge to understand the situation and find relevant hypotheses through a causal chain of reasoning.^13^

The students in the preclinical phase who had not yet developed pattern recognition (scripts) presented analytical reasoning based on their knowledge obtained from basic medical science. In contrast, students in the clinical phase used faster and non-analytical ways of reasoning with pattern recognition that included knowledge retrieval. This develops through integration of clinical knowledge, and in difficult cases, they still called upon their knowledge of basic medical science.^5^ Therefore, clinical reasoning functions as a cognitive link, through establishing a process in which knowledge of basic medical science is used as a bridge for the transition to the clinical phase.^2^

The present study, which was conducted among students, has some limitations. This study did not include the sixth-year undergraduate students because of difficulty in gaining access to them, given that they were undergoing training outside of the university’s medical school. These students would probably have higher levels of knowledge which would make the data more robust. The qualitative method is characterized by empiricism and progressive systematization until an understanding of the internal logic of a group is achieved. We sought to impose methodological rigor; however, in the data interpretation step, we might have attained only partial understanding of some of the participants’ ideas, thereby involuntarily resulting in small distortions of the data.

## CONCLUSION

In the preclinical phase of undergraduate medical education, clinical reasoning still depends on knowledge from basic medical science. In the clinical phase, when the students start to interact with patients, the pattern-recognition type of reasoning starts to develop.
